# The Roles of Monomeric GTP-Binding Proteins in Macroautophagy in *Saccharomyces cerevisiae*

**DOI:** 10.3390/ijms151018084

**Published:** 2014-10-09

**Authors:** Shu Yang, Anne G. Rosenwald

**Affiliations:** Department of Biology, Georgetown University, Washington, DC 20057, USA; E-Mail: sy265@georgetown.edu

**Keywords:** Baker’s yeast, monomeric GTP-binding proteins, macroautophagy

## Abstract

Autophagy is a cellular degradation process that sequesters components into a double-membrane structure called the autophagosome, which then fuses with the lysosome or vacuole for hydrolysis and recycling of building blocks. Bulk phase autophagy, also known as macroautophagy, controlled by specific Atg proteins, can be triggered by a variety of stresses, including starvation. Because autophagy relies extensively on membrane traffic to form the membranous structures, factors that control membrane traffic are essential for autophagy. Among these factors, the monomeric GTP-binding proteins that cycle between active and inactive conformations form an important group. In this review, we summarize the functions of the monomeric GTP-binding proteins in autophagy, especially with reference to experiments in *Saccharomyces cerevisiae*.

## 1. Autophagy in *Saccharomyces cerevisiae*

Autophagy is a process used to sequester and degrade cytoplasmic components such as proteins and organelles by utilizing a specialized membrane to form a double-membrane structure called the autophagosome, which then fuses with the lysosome [1]. Autophagy is essential for cells to survive under stressful conditions; it can serve as a way for cells to overcome starvation and is responsible for the removal of protein aggregates and damaged organelles, and for developmental remodeling. Thus, it plays an important role in controlling intracellular homeostasis [2]. Autophagy can be roughly divided into selective and non-selective forms. We will refer to the non-selective form as macroautophagy or bulk-phase autophagy. Although autophagic processes are highly conserved in eukaryotes, in this review, we will focus on the process as determined using the model organism *Saccharomyces cerevisiae*.

**Figure 1:**
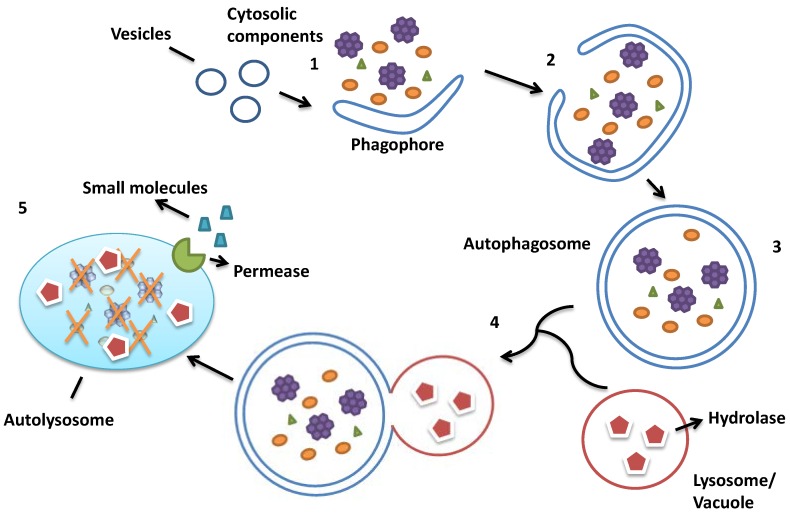
Main steps of autophagy. (1) Small vesicles fuse to form the phagophore, used to engulf the cytosolic components; (2) The expansion of the phagophore; (3) The formation of the autophagosome; (4) The fusion between the autophagosome and the lysosome; (5) The cytosolic components are digested in the autolysosome, and the resulting small molecules are released into the cytosol for reuse. (Figure is adapted from [4]).

Generally, macroautophagy starts by sensing an upstream signal, which is controlled by the Target of Rapamycin protein complex 1 (TORC1). An omega-shaped membrane structure termed the phagophore forms in the phagophore assembly site (PAS) near the lysosome (the counterpart in *S. cerevisiae* is the vacuole) and encloses cellular components such as misfolded proteins or dysfunctional organelles. The expansion of the phagophore leads to the formation of the autophagosome. After this, the autophagosome, which contains the cytoplasmic components to be degraded, fuses with the lysosome or vacuole, transferring the cargo for hydrolysis. The inner membrane as well as the enwrapped cargo is degraded and the resulting building blocks are released into the cytoplasm by lysosomal/vacuolar membrane permeases for re-use in biosynthesis (Figure 1) [3].

**Figure 2:**
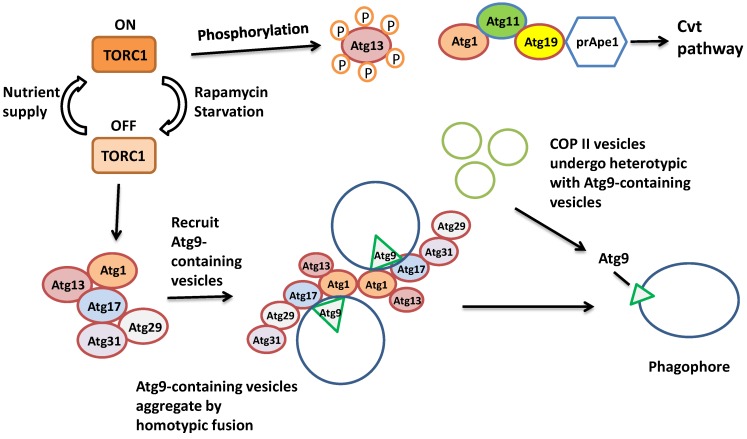
The initiation of autophagy. (1) Under nutrient-rich conditions, active TORC1 phosphorylates Atg13; the hyper-phosphorylated Atg13 cannot bind to Atg1, so Atg1 recruits Atg11 and activates the Cvt pathway; (2) Under starvation or treatment with rapamycin, the kinase function of TORC1 is inactivated, Atg13 binds to Atg1, and recruits the Atg17-Atg29-Atg31 complex; (3) The complex recruits Atg9-containing vesicles then the vesicles fuse with each other to form the phagophore. (Figure is partially adapted from [5]). Note: in this and subsequent figures, membrane bound Atg9 is represented by green triangles.

**Figure 3:**
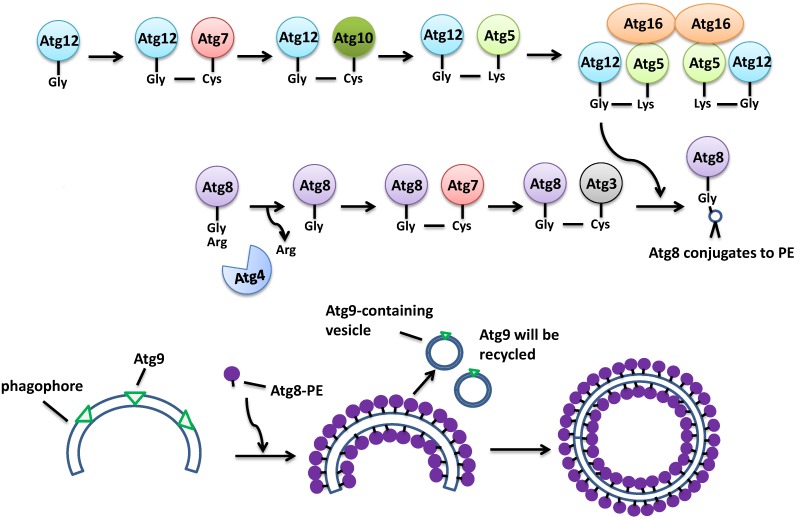
Steps to form the autophagosome. (1) Atg8 binds to PE through two ubiquitin-like pathways; (2) Atg8-PE binds to the phagophore; (3) The phagophore elongates to form the autophagosome. Atg9 recycles back to its original pool.

Induction of autophagy involves the inhibition of the TORC1 Ser/Thr kinase activity. TORC1 hyper-phosphorylates a protein called Atg13, thus inactivating it. After the inhibition of TORC1 by starvation, Atg13 becomes hypo-phosphorylated and so is activated. The active Atg13 binds to Atg1 kinase and Atg17 to form a protein complex, which will in turn recruit other proteins including Atg31 and Atg29 to serve as the platform for a number of other Atg proteins to establish the phagophore [5]. Meanwhile, Atg9 brings more membrane to help develop the phagophore (Figure 2). The initiation step of vesicle nucleation and efficient elongation requires two ubiquitin-like conjugation systems. One system involves the binding between Atg5 and Atg12 with the assistance of the E1-like Atg7 and E2-like Atg10. Then the Atg5-Atg12 complex associates with Atg16 to establish a larger protein complex, which is needed in the second ubiquitin-like pathway. The second system is involved in the covalent linkage of phosphatidylethanolamine (PE) to Atg8, also called LC3 in higher eukaryotes. Upon the protease activity of Atg4 on the *C*-terminus of Atg8, a Gly residue of Atg8 becomes exposed, then the E1-like Atg7 and E2-like Atg3, along with the Atg5-Atg12-Atg16 complex, attaches PE to Atg8, converting it into the insoluble autophagic-vesicle associated Atg8-II. At this point, Atg8 is fully activated and functions to recruit membrane for the elongation and establishment of the autophagosome (Figure 3) [6]. After the maturation of the autophagosome, it docks and fuses with the lysosome/vacuole by the mediation of a guanine nucleotide binding protein Ypt7 [7] to form the autolysosome, after which the engulfed substrates will be degraded [8].

In *S. cerevisiae*, another pathway involved in triggering autophagy is the cAMP-dependent protein kinase A pathway (PKA). Increasing the activity of the PKA pathway inhibits autophagy, while the down-regulation of this pathway leads to induction [9]. The inhibitory function of the PKA pathway is achieved by the phosphorylation of Atg13. Unlike the phosphorylation by TORC1, which makes Atg13 unable to bind to Atg1, phosphorylation of Atg13 by PKA inhibits association with the PAS thus affecting autophagy. The sites on Atg13 that are phosphorylated by TORC1 and PKA on Atg13 are distinct from one another. It is currently unknown why yeast utilizes two different triggers for autophagy. One hypothesis is that the TORC1 and the PKA pathways are both required for the cell to respond to multiple environmental nutritional conditions. Specifically, it is thought that the TORC1 senses the level of available nitrogen while the PKA pathway senses the level of carbon [10].

Although most cytosolic contents can be turned over through macroautophagy, several substrates and organelles are degraded by specific forms of autophagy. For example, in yeast, three kinds of vacuolar enzymes, the precursor forms of aminopeptidase I (prApe1) [11], α-mannosidase (prAms1) [12], and aspartyl aminopeptidase (prApe4) [13] are synthesized in the cytosol and transported to the vacuole for further processing through a selective autophagy pathway named the Cvt pathway (cytoplasm-to-vacuole targeting). The Cvt pathway is similar to macroautophagy except it happens in all conditions while macroautophagy occurs during starvation [14]. Several other forms of selective autophagy to degrade specific organelles have also been discovered, such as mitophagy for the autophagic degradation of mitochondria [15]; pexophagy for clearance of peroxisomes [16]; reticulophagy for the degradation of ER [17], and ribophagy for removal of ribosomes [18].

Research in the model eukaryote, *S. cerevisiae* has revealed the existence of more than 30 Atg proteins required for the different types of autophagy [19,20]. Besides the Atg proteins, proteins such as the soluble *N*-ethylmaleimide-sensitive fusion (NSF) protein attachment protein receptors (SNAREs) [21] and tethering factors [22,23,24] are also essential because autophagy depends intensively on membrane traffic. The monomeric GTP-binding proteins mediate multiple activities in cells, including many aspects of membrane traffic. It is therefore not surprising that they too are required in different steps of autophagy. In this review, we will summarize the functions of different kinds of monomeric GTP-binding proteins in autophagy.

## 2. The Monomeric GTP-Binding Proteins

Important processes such as cell division, protein trafficking, cytoskeleton organization, and transcription are controlled by different members of the monomeric guanine nucleotide binding protein family, including Ras, Ran, Rab, Arf/Arl/Sar and Rho [25]. The Ras sub-family regulates cell growth, survival, and division. The Ran proteins help transport RNA from the nucleus to cytosol [26]. The Rho subfamily, which includes Cdc42 and Rho proteins, is involved in cytoskeleton dynamics and cell movement [27]. The Rab proteins (usually called Ypt proteins in yeast) and Arf/Arl/Sar proteins are required in intracellular vesicle trafficking. The Rab/Ypt proteins generally function in tethering, docking, and fusion of vesicles to the target membrane, while the Arf/Arl/Sar proteins generally function in the budding of vesicles from the original membrane compartments [28,29]. These proteins are highly conserved from yeast to humans (Table 1).

**Table 1:** Membrane trafficking related monomeric GTP-binding proteins in yeast and their homologs in human.

*S. cerevisiae*	Human	Subcellular Localization	Functions in Membrane Trafficking
Ypt1	Rab1	ER-Golgi, intra-Golgi	Tethers and docks COP II vesicles to *cis*-Golgi membrane
Sar1	Sar1	ER-Golgi compartment	Assists COP II vesicles budding from ER
Arf1	Arf1	Golgi, Golgi to endosome	Regulates the budding of COP I and clathrin vesicles
Arl1	Arl1	*trans*-Golgi network (TGN)	Controls retrograde trafficking from endosome to TGN
Ypt6	Rab6	Golgi-endosome	Mediates fusion between endosome-derived vesicle to Golgi membrane
Vps21/Ypt52/Ypt53	Rab5	Early endosome	Regulates endocytic trafficking
Ypt7	Rab7	Late endosome	Regulates fusion between endosomes
Ypt31/Ypt32	Rab11	Post Golgi	Controls exocytic pathway
Sec4	Sec4	Post Golgi	Delivers exocytic vesicles to plasma membrane

**Figure 4:**
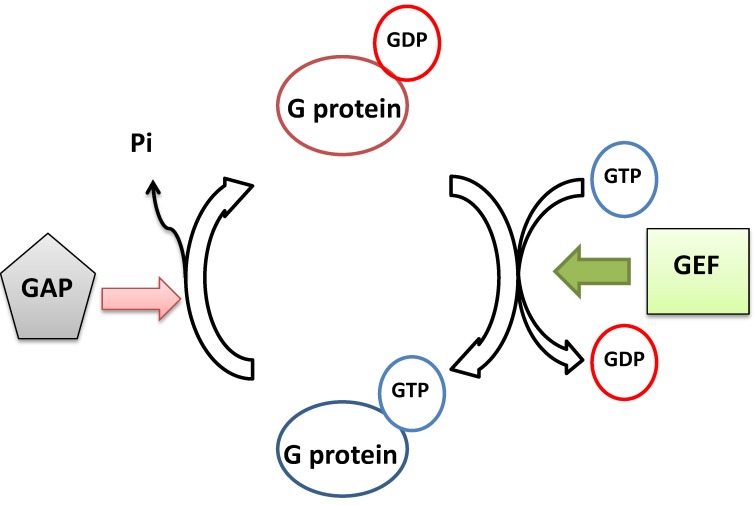
Monomeric small GTP-binding proteins can cycle between different guanine nucleotide binding states. (G protein in this figure = monomeric GTP-binding protein).

The monomeric GTP binding protein sub-families share the same functional pattern, although they are involved in different cellular processes. All of them are able to bind to GTP and GDP and can cycle between these two binding states. In their GTP-bound state, they are generally active and regulate the downstream events. When they hydrolyze GTP into GDP, they are inactivated. In general, two kinds of proteins are regulators for the G protein functional cycle. The guanine nucleotide exchange factors (GEFs) activate the monomeric G protein through exchange of GDP for GTP. On the other hand, the GTPase activating proteins (GAPs) turn off the G proteins’ function through activation of their intrinsic GTPase activity to hydrolyze GTP into GDP (Figure 4) [28]. In addition, the GDP-bound versions of Rab/Ypt proteins are controlled by another set of regulators, GDP dissociation inhibitors (GDIs) and GDI displacement factors (GDFs). Generally, Rab/Ypt proteins need to be prenylated at their *C*-terminus in order to be associated with membranes [30]. A GDI binds to the GDP-bound Rab/Ypt protein, preventing the isoprenyl side chain of the Rab/Ypt protein from binding to membrane [31] and so it remains in the cytosol. The GDF in turn sequesters the GDI and frees the GDP-bound Rab/Ypt, making it available for stimulation by its GEF protein and thus become activated. Once deactivated by its GAP, the GDP-bound Rab/Ypt will disassociate from the membrane and recycle back to the cytosol [32].

The binding of GTP to the monomeric GTP-binding proteins changes their function by affecting the three dimensional conformation. To illustrate, we will use one of the Arf family members, Arl1 (also called Arf-like 1) as an example. Arl1 functions at the *trans*-Golgi membrane and is required for membrane traffic between endosomes and the *trans*-Golgi network (TGN). In Arl1’s GDP bound form, it remains in the cytoplasm. But when it binds to GTP, it attaches to the *trans*-Golgi membrane via its *N*-terminal myristoyl group and first α-helix, and recruits its effectors to the TGN. The change in the cellular localization of Arl1 is due to movement of several secondary structures upon its binding of GTP. Like Arf proteins and other members of the superfamily, the structure of Arl1 contains domains called switch 1 (residues 40–50), switch 2 (residues 68–82) and the interswitch region (residues 51–67) [33,34]. When GTP binds to Arl1, the interswitch is displaced by a length of 2 amino acid residues from its original position. This movement makes the interswitch region occupy the hydrophobic pocket that holds Arl1’s *N*-terminal amphipathic helix, which then dislodges the helix. Finally the myristoylated N-terminus of Arl1 attaches to the membrane and participates in vesicle trafficking [35]. The deletion of *ARL1* in *S. cerevisiae* also shows a defect in K^+^ influx, suggesting Arl1 may be involved in regulating the activity of a K^+^ importer such as Trk1 [36]. Moreover, the K^+^ influx phenotype can be rescued by Arl1’s nucleotide free form, rather than its GTP bound form, suggesting a different functional cycle compared with other traditional guanine nucleotide binding proteins [37].

In recent years, it has been demonstrated that monomeric GTP-binding proteins of the Ras, Arf/Arl/Sar and Rab/Ypt protein sub-families are important for autophagy. In this review, we summarize the function of the different types of monomeric GTP-binding proteins in autophagy, specifically their roles in (1) the formation of the PAS; (2) the elongation of the PAS and the formation of the autophagosome; and (3) the trafficking of the autophgosome and the fusion between autophagosome and lysosome.

## 3. Monomeric GTP-Binding Proteins in Autophagy

### 3.1. Ras Proteins in the Early Initiation of Autophagy

As described previously, in yeast autophagy can be controlled either by the TORC1 or by the cAMP/PKA pathway, depending on the environmental cues. The yeast monomeric GTP-binding protein family member Ras2 regulates autophagy through the cAMP/PKA pathway. Ras2 and another Ras protein, Ras1 are paralogs, the result of the whole genome duplication in the evolution of yeast [38]. These two are also orthologs of proteins encoded by the mammalian *RAS* genes. Normally, the active, GTP-bound form of Ras2 localizes to the plasma membrane through docking of its *C*-terminal region, which is modified by the addition of farnesyl and palmitoyl groups [39], where it activates adenylate cyclase and increases the production of cAMP [40]. In this way, it forms the central control mechanism for metabolic rate in yeast and mediates multiple cellular activities, including sporulation, filamentous growth, and autophagy [41,42]. Generally Ras2 plays a negative role in autophagy; a hyperactive form of Ras2, namely, a mutant that cannot hydrolyze bound GTP back to GDP, can completely block autophagy, similar to the deletion of *ATG1*, a gene encoding a serine/threonine kinase required for formation of vesicles in complex with Atg13 and Atg17. (Atg1 is known as ULK1 in mammals.) Suppression of Ras2 activity leads to the induction of autophagy even under non-starvation conditions. Further, activated Ras2 affects autophagy through decreasing the number of autophagosomes in a *vam3* mutant. *VAM3* encodes a SNARE protein that mediates the fusion between the autophagosome and the lysosome. In a *vam3* mutant, since the autophagosomes cannot fuse with the lysosomes, they will accumulate in the cytosol. Because GTP-bound Ras2 decreases the number of the autophagosomes accumulating in the cytosol in this mutant background, this result suggests that GTP-bound Ras2 inhibits autophagosome formation [42].

Since the TORC1 and Ras/PKA pathways control autophagy, the main question to be determined is how these two pathways are coordinately regulated. Ras2 does not work upstream of the TORC1 because the hyperactive form of Ras2 inhibits autophagy without deactivation of TORC1 [42]. As discussed previously, PKA and TORC1 inhibit autophagy through differential effects on Atg13 phosphorylation [9]. In addition, Sch9 functions in the PKA pathway to affect autophagy. Sch9 is an ACG family (PKA, PKC, PKG) protein kinase that controls ribosome biogenesis, the activity of the Hsp90 chaperone, the initiation of translation, and the response to oxidative stress [43,44,45]. When a strain bearing a *SCH9* allele with a mutation in the ATP-binding pocket was treated with the cell permeable ATP analog, 1-(1,1-dimethylethyl)-3-(1-naphthalenylmethyl)-1*H*-pyrazolo[3,4,-d]pyrimidin-4-amine (1NM-PP1), the deactivation of Sch9 was sufficient to trigger autophagy in a TORC1-independent manner [46]. Similar results were obtained when strains with ATP-binding mutant alleles of any of the *TPK* genes, which encode paralogs of the catalytic subunit of PKA, were treated with 1NM-PP1 [46]. Sch9 and PKA may downregulate autophagy through suppression of the transcription activators Msn2/4 and Rim15 [47].

### 3.2. Monomeric GTP-Binding Proteins in the Formation of the Autophagosome

**Figure 5:**
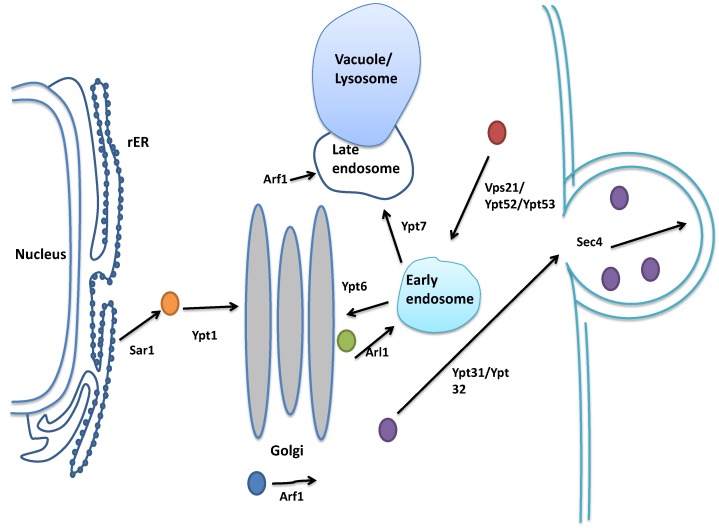
Monomeric GTP-binding proteins in vesicle trafficking in yeast.

The formation of the autophagosome requires vesicles to be delivered to the PAS. This process is mediated by several monomeric GTP-binding proteins from different secretory pathway compartments, such as Ypt1 and Sar1 from the endoplasmic reticulum (ER)-Golgi pathway, and Ypt31/Ypt32, Sec4, Arf1 and Vps21/Ypt52/Ypt53 from the Golgi-endosome compartment. Their contributions to autophagy will be reviewed below. (For the spatial localizations of the monomeric GTP-binding proteins in membrane trafficking in yeast, see Figure 5).

After the induction of autophagy, Atg9, the only known transmembrane Atg protein brings large vesicles to the PAS. Atg9 then binds to the Atg17-Atg29-Atg31 complex, and this complex induces vesicle fusion to form the omega-shaped phagophore [48]. Then the phagophore is elongated by utilizing membrane from different organelles such as the rough ER and the Golgi apparatus, to finally establish the autophagosome, the double-membrane structure that sequesters the cytosolic materials [49]. In yeast, Ypt1, the ortholog of mammalian Rab1, delivers Atg9 to the PAS. Ypt1 controls membrane trafficking events from the ER to the *cis*-Golgi as well as intra-Golgi traffic [50]. GTP-bound Ypt1 controls vesicle docking to the correct target membrane, along with specific SNARE proteins (including Uso1 [51]), to maintain the fidelity of trafficking. In addition to mediating membrane trafficking in the secretory pathway, Ypt1 plays an essential role in the formation of the phagophore. The active, GTP-bound form of Ypt1 interacts with Atg1, and this interaction can be increased by rapamycin, a drug that activates autophagy by inhibiting TORC1 [52,53]. In summary, research suggests that Ypt1 serves as a bridge to connect Atg9 and Atg1, recruiting Atg1 to the PAS and tethering the Atg9-containing vesicles together to form the phagophore [54].

Besides directly affecting autophagy through recruitment of Atg9 to the PAS, a recent study indicates Ypt1 may work with Ypt6, orthologous to Rab proteins Rab6A and Rab6B in mammals, to mediate autophagy. Ypt6 is required for the fusion between endosome-derived vesicles with *trans*-Golgi membranes [55]. Overexpression of *YPT1* rescues the autophagic defect exhibited in yeast strains lacking *YPT6* through increasing the amount of Atg8 sorted correctly to the vacuole. Thus Ypt1 functions downstream of Ypt6 [56] but the detailed mechanism for the relationship between these two Rab proteins in autophagy needs further investigation.

Ypt1 can either mediate membrane trafficking events in the ER-Golgi compartment or in the PAS. The spatial and functional distinction for this protein’s activity is achieved by the transport protein particle (TRAPP) complex, which also acts as the Ypt1 GEF. The TRAPP complex has three subtypes and all three share seven core subunits that are conserved from yeast to humans. The seven core subunits (Bet3A, Bet3B, Bet5, Trs20, Trs23, Trs31, Trs33) form the basic structure called TRAPP I, and it contains the GEF activity provided by Bet3A, Bet3B, Bet5, Trs23, and Trs31 responsible for activating Ypt1 and controlling traffic in the ER-Golgi compartment [57]. The intra-Golgi and Golgi-endosome trafficking is mediated by TRAPP II, which contains another three subunits: Trs65, Trs120 and Trs130 in addition to the 7 TRAPP I subunits. Studies have suggested the TRAPP II complex can either act as the GEF for Ypt1 in the intra-Golgi region, or for other Rab family members, the paralogous pair Ypt31/Ypt32 for Golgi-endosome trafficking [58] but this hypothesis is still controversial, as others believe that TRAPP II only acts as the GEF for Ypt1 in the intra-Golgi trafficking [59]. Autophagy is controlled by the TRAPP III complex, which is TRAPP I with the addition of one additional subunit, Trs85. Trs85 helps localize the TRAPP III complex to the PAS through interactions with Atg17, thus recruiting Ypt1 to the PAS [23]. The distinct structure and localization of the TRAPP complex ensures that Ypt1 works in specific compartments of the cell.

Another monomeric GTP-binding protein in the early secretory pathway that affects the formation of the autophagosome is Sar1, a member of the Arf/Arl/Sar sub-family that interacts with the COP II-related Sec proteins. Upon activation of Sar1 by Sec12, its GEF, it binds to the ER membrane through its *N*-terminal myristoyl modification. The ER-bound Sar1 generates membrane curvature and recruits the heterodimer pairs Sec23-Sec24 and Sec13-Sec31, coat proteins that form the COP II vesicle, which will then bud from the donor membrane. Yeast lacking *SEC12* are defective in the formation of the autophagosome, and this defect is rescued by the overexpression of *SAR1*. Also strains with temperature-sensitive alleles of *SEC23* or *SEC24* have decreased autophagy at the restrictive temperature [60]. These results indicate the possibility that COP II vesicles are required in autophagy. Recent research using single-particle electron microscopy has revealed that the TRAPP III complex can also recruit COP II vesicles to the PAS through binding to Sec23. Therefore, the Sar1/COP II system can provide some of the membrane resources necessary to form the autophagosome [61]

Other monomeric GTP-binding proteins that localize to the post-Golgi compartment are also essential in autophagy, including Sec4 and Ypt1/Ypt32 members of the Rab protein family. Sec4 mediates transport of vesicles from the *trans*-Golgi to the plasma membrane, which is the last step of the exocytic secretory pathway [62]. In Sec4’s GTP bound state, it can recruit effectors such as one of the subunits of the exocyst complex, Sec15 [63], resulting in fusion of vesicles with the plasma membrane, extruding the contents of the lumen to the exterior of the cell. Sec4 along with its GEF, Sec2, are required in autophagy. Lacking the function of either one leads to decreased levels of autophagy at least in part because of a defect in the trafficking of Atg9 to the PAS. Atg9 travels between PAS and the Atg9 reservoir [64] bringing vesicles to the PAS and helping to form the autophagosome, so the number of Atg9-attached vesicles reaching the PAS can affect the ultimate number of autophagosomes. In strains lacking *SEC2* or *SEC4*, the amount of Atg9 that colocalizes with the PAS marker Ape1 decreases, and the number of autophagosomes is reduced, suggesting that Sec2 and Sec4 are required for the correct localization of Atg9 to the PAS [65].

Other monomeric GTP-binding proteins in the post-Golgi compartment also contribute to delivery of vesicles to the PAS, including the Rab family members, Ypt31 and Ypt32, paralogs that resulted from the whole genome duplication [66]. Generally, Ypt31/Ypt32 is required for vesicles to exit from the TGN, and recruit Sec2 and Sec4 to the budding vesicle [67]. Deletion of these two *SEC* genes leads to decreased autophagic activity [65]. Moreover, when GFP-labeled Atg8 was used to visualize autophagosome morphology [68], a multiple punctate phenotype was observed in a *ypt31Δ/32ts* strain at the restrictive temperature, suggesting that these GFP-Atg8-containing autophagosomes cannot enter the vacuole. Atg8 seems to accumulate in abnormal locations rather than at the PAS. Therefore, the activity Ypt31/Ypt32 is essential in forming the autophagosome by affecting the correct localization of Atg8 to the PAS [65].

Two Rab family members in the post Golgi compartment that contribute to autophagy are Vps21 (or Ypt51) and Ypt52, orthologs of mammalian Rab5s. Vps21 and Ypt52 along with Ypt53, another paralog of Vps21 are monomeric GTP binding proteins in the endosome system and are required for endocytic transport and the correct localization of the multimeric tethering complex called CORVET (class C core vacuole/endosome tethering) to the endosome [69,70]. Studies show that Vps21 works with Ypt52 and both are required for efficient autophagy. Deletion of both genes decreases the intra-vacuolar accumulation of Atg8 [71]. Moreover, a recent study shows Vps21/Ypt52/Ypt53 may function before the fusion between autophagosomes and vacuole [72].

Besides the Rab proteins, members of the Arf/Arl/Sar family that function in the post-Golgi compartment are also involved in autophagy. Arf1, ADP-ribosylation factor 1, participates in the formation of coated vesicles from the *trans*-Golgi membrane; this function of Arf1 is similar to Sar1. These vesicles bring cargo through the exocytic or endocytic pathway to the target membrane. Along with its GEF Sec7, Arf1 is essential for the efficient expansion of the PAS to form the autophagosome membrane, although in *sec7* or *arf1* mutant strains, the ability to establish the PAS is intact [73]. Similar to the function of Sec4-Sec2 in autophagy, Arf1-Sec7 affects the anterograde trafficking of Atg9 to the PAS. Although Arf1 is required in the formation of COP I and clathrin-coated vesicles, neither a defect in COP I nor clathrin-coated vesicles (CCVs) shows an autophagic defect in yeast [73], suggesting that Arf1 and Sec7 affect autophagy through regulating effectors other than COP I and CCVs. Nevertheless, the research above suggests that macroautophagy requires membranes from different organelle compartments in cells. Blocking one of these pathways for bringing membrane to the growing PAS inhibits the formation of autophagosomes, affecting either size or number, and thus decreasing the amount of autophagy.

### 3.3. Monomeric GTP-Binding Proteins in the Fusion between the Autophagosome and the Vacuole

After the expansion and formation of the autophagosome, autophagy enters its later steps. The autophagosome, which has engulfed the cytosolic components, fuses with the vacuole to form the autolysosome. One monomeric GTP-binding protein that is essential in this process is Ypt7.

Ypt7 orthologous to mammalian Rab7 controls the trafficking in the late endosome compartment by mediating homotypic fusion between endosomes and heterotypic fusion between late endosomes and the vacuole [7,74]. Ypt7 is activated by its GEF, the Ccz1-Mon1 complex [75]. Once activated, GTP-bound Ypt7 inserts into the membrane of both the late endosome and the vacuole. The GTP-bound Ypt7 then recruits the homotypic fusion and vacuole protein sorting (HOPS) tethering complex to late endosomes containing the SNARE protein, Vam7, through binding to the Vps39 and Vps41 subunits of HOPS [76]. HOPS orients the late endosome near the vacuole. Then the target SNARE Vam7 on the late endosome binds to Vam3 to form a tSNARE complex and direct fusion [77]. The Vam7-Vam3 tSNARE complex binds to its vesicle SNARE partner Nyv1, localized on the vacuole membrane. The resulting *cis*-SNARE complex directs the fusion between late endosome and the vacuole [78]. In autophagy, Ypt7 is required for the fusion between the autophagosome and the vacuole. The mechanism is similar to the general vesicle fusion with the vacuole. The deletion of *YPT7* and the disruption of Vam7/Vam3 as well as the HOPS complex under starvation conditions cause the accumulation of autophagosomes in the cytoplasm [79,80], demonstrating the defect in autophagosome and vacuole fusion.

## 4. Summary and Conclusions; Future Directions

**Figure 6:**
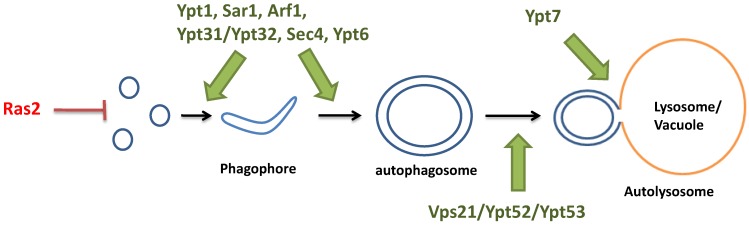
Summary of the known functions of the monomeric GTP-binding proteins in autophagy. (1) Ras2 inhibits the initiation of autophagy; (2) Ypt1, Sar1, Arf1, Ypt31/Ypt32, Sec4, and Ypt6 are required for phagophore formation and expansion to form the autophagosome; (3) Vps21/Ypt52/Ypt53 are required for the efficient localization of Atg8 to the vacuole; and (4) Ypt7 is required for the fusion between autophagosome and vacuole.

In this review, we have summarized the roles of different monomeric GTP-binding proteins and their regulators and effectors in each step of macroautophagy in *S. cerevisiae*, from the initiation of autophagy, the formation of autophagosome, to the fusion between autophagosome and vacuole. To summarize, Ras2 from the Ras family controls the cAMP/PKA pathway which is one of the two pathways that triggers autophagy in yeast; Ypt1 from the Rab family is responsible for bringing Atg9-containing vesicles to the PAS for the formation of autophagosome; Sar1 from the Arf/Arl/Sar family brings COP II vesicles for the expansion of autophagosome; Sec4 and Arf1 in the post-Golgi compartment control anterograde trafficking of Atg9 to the PAS; Ypt31/Ypt32 mediates the correct localization of Atg8 to the PAS; and Vps21/Ypt52/Ypt53 regulates the efficient localization of Atg8 to the endosomal system. In the final steps of autophagy, Ypt7 is required for fusion of the autophagosome to the vacuole (summarized in Figure 6).

Despite the advances in understanding how monomeric GTP-binding proteins regulate autophagy, questions still remain. The first question is how many of the known monomeric GTP-binding proteins have roles in autophagy. To date, many monomeric GTP-binding proteins have been shown to have multiple functions, particularly those that control membrane traffic, but the list of functions is expanding. For example, Arl1 may have a role in autophagy. Normally Arl1 mediates vesicle trafficking between endosomes to the *trans*-Golgi and is required for vacuole-targeting transport [81], but Arl1 is also required for a form of cell death referred to as autophagic cell death in yeast [82]. How autophagic cell death relates to macroautophagy is presently unclear. Another question is how the monomeric GTP-binding proteins are transferred from the compartments in which they act in membrane traffic to the locations for autophagy such as the PAS. The data to date suggest that there may be at least two functional models. One is exemplified by Ypt1, which in the presence of TRAPP III directs COP II vesicles to the phagophore [61]. Another is exemplified by Arf1, which may contribute to autophagy through controlling other kinds of vesicles or factors that are important to autophagy since it appears that loss of the ability to create COP I and clathrin-coated vesicles has no effect on macroautophagy, yet loss of Arf1 adversely affects the process. Moreover, most research efforts focus on monomeric GTP-binding proteins in macroautophagy and the Cvt pathway, but there is little information about which monomeric GTP-binding proteins are involved in other kinds of selective autophagy such as mitophagy and pexophagy. In addition, the subtle differences in membrane trafficking remain to be explored. As an example, selective autophagy in yeast requires the actin cytoskeleton for correct transfer of specific cargo such as the prApe1 to the PAS, while in macroautophagy the actin cytoskeleton does not appear to be involved [83].

Autophagy is a complicated process, requiring the participation of protein factors from multiple pathways. Because monomeric GTP-binding proteins act as coordinators of many cellular activities, especially membrane traffic, these proteins are essential in all steps of autophagy. The relationship between monomeric GTP-binding proteins and autophagy can help to link the protein interaction network underlying autophagy with other cellular processes such as the secretory pathway, endocytosis, and cell growth.
